# Advancing palliative care in cardiac arrest and cardiogenic shock: identifying evidence gaps and future research priorities

**DOI:** 10.3389/fcvm.2026.1845595

**Published:** 2026-06-03

**Authors:** Anne Song, Oscar J. L. Mitchell, Jacopo D’Andria Ursoleo, Donald R. Sullivan, William E. Rosa, Katherine R. Courtright, Rachel Kohn

**Affiliations:** 1 Department of Medicine, Perelman School of Medicine at the University of Pennsylvania, Philadelphia, PA, United States; 2 Palliative and Advanced Illness Research (PAIR) Center, Perelman School of Medicine at the University of Pennsylvania, Philadelphia, PA, United States; 3 Leonard Davis Institute of Health Economics, Perelman School of Medicine at the University of Pennsylvania, Philadelphia, PA, United States; 4 Center for Resuscitation Science, Department of Emergency Medicine, University of Pennsylvania, Philadelphia, PA, United States; 5 Department of Anesthesia and Intensive Care, IRCCS San Raffaele Scientific Institute, Milan, Italy; 6 Department of Medicine, Division of Pulmonary, Allergy, and Critical Care Medicine, Oregon Health & Science University, Portland, OR, United States; 7 Department of Psychiatry and Behavioral Sciences, Memorial Sloan Kettering Cancer Center, New York, New York, United States

**Keywords:** cardiac arrest, cardiogenic shock, cardiovascular diseases, critical illness, health services, palliative care, patient-centered care

## Abstract

**Introduction:**

Cardiac arrest and cardiogenic shock are life-threatening cardiovascular emergencies that impose substantial physical, psychological, and decisional burdens on patients and caregivers. Palliative care, defined as holistic, person-centered care focused on preventing and relieving symptoms and stressors of serious illnesses to improve quality of life for patients and caregivers, is increasingly recognized as an essential component of comprehensive cardiovascular care.

**Objectives:**

We therefore conducted a narrative review to synthesize current evidence regarding inpatient palliative care in cardiac arrest and cardiogenic shock; identify critical gaps in research and practice; and propose future directions to guide clinicians, researchers, and policymakers, while centering patients and caregivers.

**Discussion:**

In the U.S., palliative care utilization for patients with cardiac arrest and cardiogenic shock has increased significantly over the last two decades. However, the literature on palliative care in cardiac arrest and cardiogenic shock is limited, demonstrating that palliative care consultation is associated with increased do-not-resuscitate orders and withdrawal of life-sustaining treatments, as well as decreased healthcare utilization and subsequent costs. However, patient- and caregiver-centered outcomes have not been studied. Major palliative care research gaps exist in measurement, equity, education, and delivery models, including the optimal roles of generalist vs. specialist palliative care. Future research should prioritize patient- and caregiver-centered outcomes, comparative effectiveness of different palliative care delivery models, implementation science, health equity, and education and training of generalist palliative care skills to optimize palliative care integration for cardiac arrest and cardiogenic shock to improve the lived experience of these patients and their caregivers.

## 1 Introduction

Cardiac arrest and cardiogenic shock represent two of the most acute, life-threatening cardiovascular emergencies. Cardiac arrest is characterized by sudden cessation of cardiac function. In the United States (U.S.), out-of-hospital cardiac arrest (OHCA) occurs in ∼350,000 adults annually, with survival rates of ∼10%–12% (1, 2). In-hospital cardiac arrest (IHCA) affects an additional 290,000 U.S. adults annually, with survival to hospital discharge in ∼25% (3). Globally, the burden is similar, with 4–6 million individuals affected annually with varying incidence across continents and healthcare systems (4–9). Cardiogenic shock, a state of severely impaired tissue perfusion due to cardiac dysfunction, most commonly results from acute myocardial infarction or decompensated heart failure, complicating 5%–10% of such hospitalizations (10–14). Despite advances in mechanical circulatory support and acute interventions, cardiogenic shock continues to cause significant morbidity and mortality, with in-hospital mortality exceeding 30%–40% depending on etiology, severity, and treatment availability (10, 14).

Both conditions impose extraordinary burdens on patients and caregivers. For cardiac arrest survivors, the post-arrest period in the intensive care unit (ICU) is often characterized by neurological uncertainty, the need for surrogate decision-making, and high risk of post-intensive care syndrome among patients and caregivers (15–19). Survivors often experience long-term physical, psychological, and cognitive sequelae that profoundly impact quality of life (QOL) and functional recovery. Patients with cardiogenic shock similarly experience significant symptom burdens, including dyspnea, fatigue, pain, and psychological distress, compounded by the uncertainty inherent in critical illness and the potential need for advanced and invasive therapies such as mechanical circulatory support or heart transplantation (20–23).

Palliative care, defined as holistic, person-centered care focused on preventing and relieving symptoms and stressors of serious illnesses to improve QOL for patients and caregivers (24, 25), is increasingly recognized as an essential component of comprehensive cardiovascular care (26). Unlike hospice care, which is reserved for patients in the last 6 months of life, palliative care can be provided alongside curative therapies and at any stage of illness. It encompasses both generalist (or primary) palliative care, delivered by all clinicians as part of routine care, and specialist (or secondary) palliative care, provided by interdisciplinary teams with subspecialty training in complex symptom management, psychosocial support, and serious illness communication (27).

The American Heart Association (AHA) recently released scientific guidelines emphasizing the importance of palliative care integration in critical cardiovascular illness (26). These guidelines, along with other recent reviews (28–30), highlight the current state of palliative care in the cardiac ICU. To augment this growing literature, our aims in this narrative review were to synthesize current evidence regarding inpatient palliative care in cardiac arrest and cardiogenic shock; identify critical gaps in research and practice; and propose future directions to guide clinicians, researchers, and policymakers, while centering patients and caregivers. Outpatient integration of palliative care before or after cardiac arrest and cardiogenic shock is outside of the scope of this review, though earlier integration of palliative care in chronic serious illnesses (e.g., heart failure) that increase the risk of cardiac arrest and cardiogenic shock is recommended (31, 32). As there has not been widespread adoption of this recommendation, and because cardiac arrest and cardiogenic shock may be the initial presentation of life-limiting illnesses, we will focus on the inpatient setting. Furthermore, we recognize that differences in policy, culture, and resources may influence palliative care delivery in different countries (33). While we have cited international literature where available, this review is primarily based on U.S. studies given the literature base.

## 2 Utilization patterns, trends, and disparities

In the U.S., palliative care utilization for patients with cardiac arrest has increased significantly over the last two decades, from 1.5% of OHCA hospitalizations in 2002 to 19.6% in 2016–2021 (34, 35), with similar trends across common OHCA etiologies (e.g., acute myocardial infarction, pulmonary embolism) (36, 37). In cardiogenic shock, palliative care utilization has also increased from <2% in the early 2000s among patients with acute myocardial infarction (38, 39) to 21.9% of hospitalizations in 2020 nationally (40, 41), with trends mirrored across etiologies and severity (42).

These studies also report disparities in palliative care delivery (i.e., differences in palliative care receipt by sociodemographic characteristics unrelated to disease severity or clinical need) among both cardiac arrest and cardiogenic shock populations, with older age, female gender, White race, higher comorbidity burden, higher socioeconomic status, and admission to larger hospitals associated with higher receipt (34–38, 42, 43). However, these national studies rely on administrative data and billing codes [V66.7 [ICD-9-CM], Z51.5 (ICD-10-CM)] to identify palliative care delivery, which have low sensitivity and positive predictive value for specialist palliative care (44), limiting interpretability.

## 3 Outcomes and benefits of palliative care

While the theoretical benefits of palliative care in cardiac arrest and cardiogenic shock are compelling, empirical evidence specific to these conditions remains limited. Most knowledge is extrapolated from heart failure (45–48) and critical care (49, 50), including trauma (51, 52) and severe acute brain injury (SABI) populations (53, 54).

### 3.1 Evidence from heart failure

Among heart failure populations, palliative care has been associated with improvements in physical and psychological symptom burden (dyspnea, pain, fatigue, depression, anxiety), spiritual well-being, QOL, functional class, patient and caregiver satisfaction, more comprehensive advance care planning, increased hospice utilization, and reduced healthcare costs through decreased hospital and ICU admissions and lengths of stay (47, 55–63). Notably, the generalist and specialist palliative care interventions studied have spanned inpatient, outpatient, transitional, and home care settings. Thus, guidelines across major cardiology societies recommend that palliative care be integrated in routine heart failure care not only for those with advanced disease, but for all patients with heart failure, regardless of severity (31, 32).

### 3.2 Evidence from critical care, trauma, and severe acute brain injury (SABI)

Within critical care settings, various generalist and specialist palliative care interventions have been studied (49, 64, 65), demonstrating associations with improved caregiver satisfaction with communication and overall care (66, 67), improved clinician satisfaction and burnout (68–70), reduced hospital and ICU lengths of stay (66–68, 71–73), and reduced care costs (72–74). Some interventions have also demonstrated improvements in post-ICU caregiver psychological distress (72, 75–78), but these results have been mixed, likely reflecting the heterogeneity of interventions, methods, and critical illness (66, 79, 80). Post-ICU psychological outcomes may be particularly challenging to improve due to the multifactorial and prolonged nature of psychological distress, particularly among bereaved caregivers (81, 82), and limitations of measurement and intervention timing (83). Incorporating palliative care practices in ICUs is widely recommended by professional palliative care and critical care societies (50, 84–88).

Evidence from trauma and SABI may be particularly informative for OHCA populations, as they often involve sudden transitions from wellness to critical illness. Palliative care can support caregivers navigating unexpected tragedy, short- and long-term prognostic uncertainty, and surrogate decision-making. Integration of early generalist and specialist palliative care (i.e., ≤3 days after admission) in trauma populations has been associated with reduced lengths of stay, rates of invasive procedures, and care costs compared to late consultation, though the impact on patient- and caregiver-centered outcomes remains largely unknown (89–93). For patients with SABI due to stroke, traumatic brain injury, and hypoxic-ischemic injury (including from cardiac arrest), surrogate decision-makers often experience severe and persistent psychological distress (94). Thus, recent studies have focused on the palliative needs of this population and how improved communication may better support them in the face of extended prognostic uncertainty and grief (95–100).

### 3.3 Evidence from cardiac arrest and cardiogenic shock

For cardiac arrest and cardiogenic shock specifically, studies have shown that palliative care consultation is associated with increased do-not-resuscitate orders and withdrawal of life-sustaining treatments, as well as decreased healthcare utilization and subsequent costs (35–38, 40–42, 71, 101–103). However, patient- and caregiver-centered outcomes have not been studied.

## 4 Discussion

**Figure 1:**
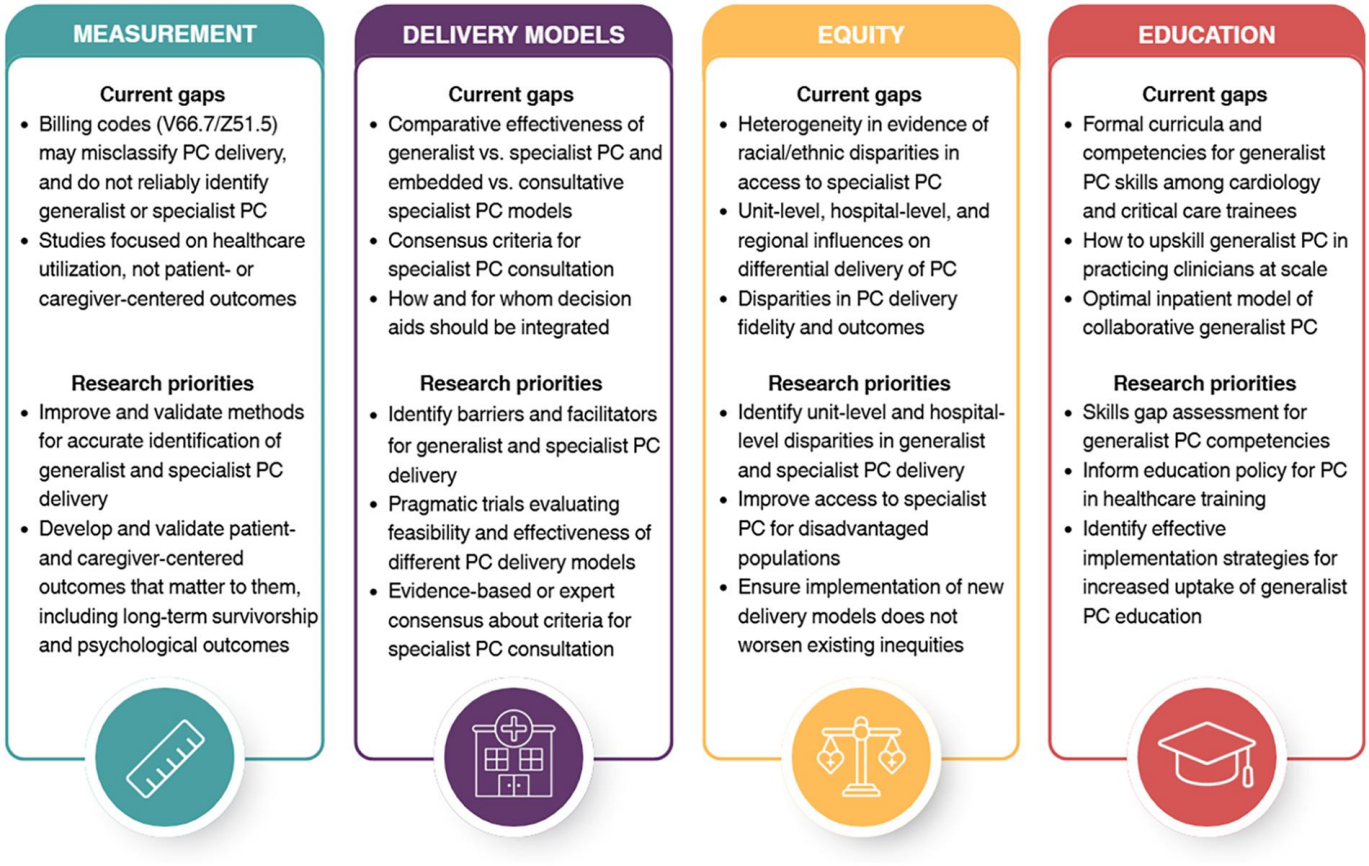
Evidence gaps and research priorities for delivering palliative care in cardiac arrest and cardiogenic shock. To advance the delivery of palliative care, current evidence gaps and research priorities are organized across four domains of measurement, delivery models, equity, and education. PC, palliative care.

Despite growing recognition of palliative care's importance for patients experiencing cardiac arrest and cardiogenic shock and their caregivers, substantial gaps persist in research, practice, and policy (Figure 1). While extrapolation from heart failure, critical care, trauma, and SABI literature provides guidance, major questions remain about whether these conditions require tailored palliative care delivery approaches.

### 4.1 Measurement gaps in palliative care research

The current evidence base for generalist or specialist palliative care in cardiac arrest and cardiogenic shock is characterized by considerable limitations. Most existing studies have focused on utilization patterns and care processes, rather than on patient- and caregiver-centered outcomes (34–43, 101, 102). Furthermore, data quality regarding access to and disparities in palliative care utilization remain a concern. Studies using administrative data and billing codes to identify palliative care delivery have limitations in their ability to distinguish the delivery of generalist or specialist palliative care from comfort-focused end-of-life care (44, 104–106). Beyond concerns about misclassification, these studies also cannot assess the quality, intensity, or effectiveness of the care delivered. Future research must move beyond healthcare utilization metrics to evaluate outcomes that matter to patients and caregivers (83). For example, QOL, care satisfaction, alignment between care received and patient goals and values (i.e., goal-concordant care), psychological distress, and long-term recovery trajectories (e.g., physical, psychological, cognitive) among survivors represent just some of the meaningful but understudied outcomes in these conditions.

### 4.2 Unique considerations in cardiac arrest

In patients with cardiac arrest, neurological prognostication following resuscitation can be characterized by profound uncertainty. Guidelines recommend waiting ≥72 h after return of spontaneous circulation—often longer with targeted temperature management—before providing prognostication (107). Thus, caregivers face both acute crisis and prolonged ambiguity, which can extend far beyond this initial 72-hour window given the timeframes for potential recovery (54, 97, 98).

How palliative care can facilitate prognostication and communication, and whether this should be delivered through generalist- or specialist-dominant models, remains unresolved. Innovative programs such as RECOVER (Recovery of Consciousness via Evidence-Based Medicine and Research) at the Hospital of the University of Pennsylvania integrate specialist palliative care early and longitudinally in post-arrest care (108). Whether this is optimal or scalable requires further study, particularly in palliative care resource-limited settings, such as community or rural hospitals.

Important distinctions also exist between OHCA and IHCA. OHCA is typically unexpected and may occur without prior advance care planning, placing caregivers in the position of making decisions without the patient's guidance (109, 110) In contrast, IHCA typically occurs in older patients with more comorbidities who are already admitted to the hospital, often with acutely or chronically life-threatening illnesses, allowing opportunities for anticipatory discussions and caregiver preparation for surrogate decision-making (111). These differences may warrant distinct communication strategies and indicators for consultation.

### 4.3 Unique considerations in cardiogenic shock

Cardiogenic shock often requires time-sensitive decisions regarding mechanical circulatory support and advanced therapies, including intra-aortic balloon pumps, extracorporeal membrane oxygenation, ventricular assist devices, and transplantation. Decision aids developed for ventricular assist device decision-making may help structure discussions and align choices with patient values (112–114). However, their routine integration into acute shock care remains understudied (115, 116). Specialized heart failure and mechanical circulatory support teams may provide targeted opportunities to embed generalist palliative care skills.

### 4.4 Models for integration

**Figure 2:**
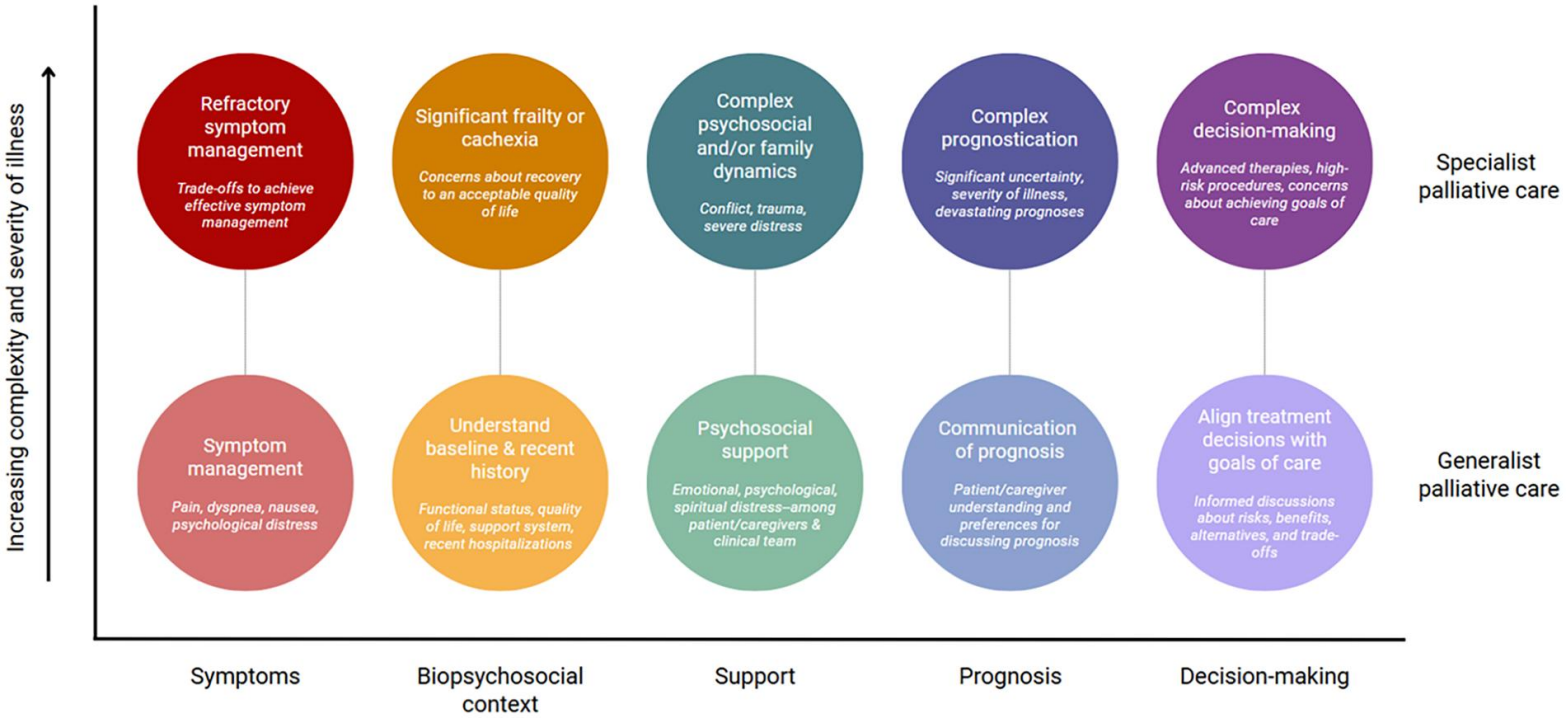
Hypothetical indications for delivery of generalist and specialist palliative care in cardiac arrest and cardiogenic shock. The *y*-axis denotes increasing medical or psychosocial complexity and severity of illness; the *x*-axis denotes various domains of palliative care. The bottom row identifies palliative care delivery consistent with proposed generalist palliative care competencies for the cardiovascular specialist (21). The top row identifies potential criteria for specialist palliative care consultation, though the threshold to consult specialist palliative care may move up and down for any individual domain, depending on comorbid palliative care needs as well as the bandwidth and comfort of the primary team in providing palliative care. This figure is adapted from Sullivan DR, et al. Palliative Care Early in the Care Continuum among Patients with Serious Respiratory Illness: An Official ATS/AAHPM/HPNA/SWHPN Policy Statement. *Am J Respir Crit Care Med*. 2022;206(6):e44–e69, with permission from the American Thoracic Society.

A fundamental question facing the field is how palliative care should be delivered in cardiac arrest and cardiogenic shock. While direct comparisons are limited, generalist palliative care is likely less effective than specialist palliative care in some serious illnesses (117). However, ongoing shortages in the specialist palliative care workforce require more urgent investigation of where and how generalist palliative care may most effectively meet the needs of these populations and their families (118–120). Additionally, geographic differences in specialist palliative care availability may influence the optimal strategies (121). Embedded models, in which specialist palliative care teams participate in multidisciplinary rounds alongside cardiology and critical care teams, may facilitate early identification of needs and collaborative decision-making, but require sufficient workforce capacity (122, 123). Hybrid approaches combining generalist palliative care with selective specialist consultation may be pragmatic, but require clear criteria for consultation, training, and quality oversight (Figure 2) (88, 124). Studies of comparative effectiveness of palliative care delivery models in cardiac arrest and cardiogenic shock populations are urgently needed, particularly across settings with varying levels of resources.

### 4.5 Identifying indicators for specialist consultation

Cardiac arrest has been proposed as an automatic indication for palliative care consultation (125). Whether universal consultation represents optimal use of limited resources remains uncertain. Some patients may benefit equally from generalist palliative care, whereas others facing more complex prognostic, psychosocial, or ethical challenges may warrant specialist involvement (Figure 2). Developing evidence-based and scalable criteria (126) or at least expert consensus criteria—similar to Delphi consensus criteria for advanced heart failure (47)—could better target specialist care to those most likely to benefit from it. Such criteria should be prospectively validated and evaluated for unintended consequences, including worsening of existing disparities.

A similar question exists for cardiogenic shock, for which relevant policy already exists. Since 2013, the U.S. Centers for Medicare and Medicaid Services has mandated that all patients undergoing evaluation for durable left ventricular assist device implantation have access to palliative care prior to surgery (127, 128). Subsequently, many programs instituted automatic pre-implantation palliative care consults, though there has been variability in interpretation (129–131). Again, whether universal consultation is necessary or beneficial for all patients or whether generalist palliative care may be sufficient for some patients, particularly if decision aids are incorporated into routine care, warrants further investigation.

### 4.6 Generalist palliative care education and training

Strengthening generalist palliative care competencies among cardiology and critical care clinicians is essential (132). A 2016 national survey found cardiology trainees valued palliative care, but felt insufficiently prepared (133). This led the AHA to call for improved training in residency and fellowship programs, including palliative care competencies for cardiovascular specialists (26). Similar deficiencies exist in critical care training programs (134). Addressing this gap likely requires systematic curricula, simulation, and competency assessments. Improving the generalist palliative care skills for clinicians caring for these patients across all disciplines could also lead to a multi-disciplinary, collaborative team approach to generalist palliative care delivery, which would enhance scalability and overcome specialist palliative care workforce shortages (59, 60). Additionally, continuing medical education and quality improvement initiatives could help practicing clinicians develop and maintain these skills.

### 4.7 Precision palliative care and equity

Because specialist palliative care is a limited resource, “precision specialist palliative care” has emerged as a promising framework for targeting those most likely to benefit. However, ensuring that new models do not exacerbate inequities is essential.

Disparities in specialist palliative care receipt by race and ethnicity are heterogeneous across studies. In the U.S., smaller cohort studies using electronic health record (EHR) data do not identify the same disparities that larger, national studies using administrative data do (101, 103). In addition to hospital-level heterogeneity in palliative care delivery (135), these discrepancies are likely driven by key methodologic differences, including how specialist palliative care delivery was identified [e.g., EHR data vs. flawed administrative billing codes (44)]; where data were collected (e.g., single-center vs. multi-center aggregated data that may mask trends in specific groups when these groups are combined, i.e., Simpson's paradox); and geographic location (e.g., urban vs. rural vs. combined hospital samples). While it is possible that racial and ethnic minorities may be systematically underserved by palliative care, more rigorous research is needed to answer this question in general, and specifically among patients with cardiac arrest and cardiogenic shock (44, 104–106).

The evidence is clearer that rural, unhoused, and surgical patients experience decreased access to specialist palliative care (136–138). As of 2024, 96.2% of large U.S. hospitals with >300 beds reported specialist palliative care services, compared to 34.5% of rural hospitals (121, 136). This may be particularly pertinent for patients who are less likely to be transferred to tertiary or quaternary care centers for interventions and advanced therapies. Unhoused populations experience increased barriers to accessing healthcare across specialties, including palliative care (137, 139). Given the increased risk of OHCA among unhoused patients (140), this warrants attention. Additionally, individuals experiencing homelessness may have different needs or challenges regarding advance care planning, surrogate decision-maker identification, and post-discharge support. Palliative care consultation rates also differ by medical vs. surgical service, suggesting a broader ingrained cultural barrier within some fields may play an important role in specialist palliative care delivery beyond known unit- and clinician-level heterogeneity (138, 141). These patients, including those undergoing cardiac surgery who subsequently experience cardiac arrest or cardiogenic shock, may have different but equally burdensome palliative needs than their medical counterparts, yet have received less attention in research and practice.

Importantly, disparities may extend beyond access to specialist palliative care consultation. Understanding disparities in generalist palliative care delivery, fidelity to high-quality generalist or specialist palliative care, and outcomes remain pressing research priorities.

### 4.8 Specialist palliative care team composition and workflow

Optimal team composition and workflow for specialist palliative care in cardiac arrest and cardiogenic shock require further study. Interdisciplinary palliative care teams may include physicians, advanced practice providers, nurses, social workers, chaplains, psychologists, and pharmacists among others, each addressing distinct patient and caregiver needs (84). However, specialist palliative care staffing is highly variable across U.S. hospitals, with few meeting national guidelines, and it is not yet understood how team composition may affect palliative care quality or outcomes (142, 143). Optimizing team composition, triage, and resource allocation remains an important area for further investigation.

### 4.9 Future research priorities

Advancing palliative care in cardiac arrest and cardiogenic shock will require a comprehensive research agenda addressing multiple domains (Figure 1). In addition to improving the accuracy of identification and reporting of palliative care delivery, priority areas include:

1. **Patient- and caregiver-centered outcomes:** Moving beyond utilization metrics to examine QOL, symptom burden, psychological outcomes, patient and caregiver satisfaction, and goal-concordant care, including validating appropriate measurement tools.
2. **Comparative effectiveness:** Evaluating different delivery models (generalist vs. specialist; embedded vs. consultative specialist care; criteria-based vs. universal consultation) to identify which approaches are most effective, efficient, and equitable, and for whom.
3. **Implementation science:** Describing the multi-level barriers and facilitators to generalist and specialist palliative care delivery in cardiac arrest and cardiogenic shock care, developing tailored implementation strategies, and evaluating applicability and scalability of successful models.
4. **Health equity:** Investigating mechanisms underlying disparities in access to, fidelity of, and outcomes of generalist and specialist palliative care; testing equity-promoting interventions; and ensuring new models do not worsen inequities.
5. **Education and training:** Developing and evaluating generalist palliative care curricula for cardiology and critical care trainees and clinicians, with validated competency assessment.
6. **Decision-making support tools:** Adapting existing or developing and validating new decision aids for cardiac arrest (neurological prognostication, treatment escalation/de-escalation) and cardiogenic shock (mechanical circulatory support) and studying their impact on decision quality and patient-centeredness.

## 5 Conclusion

Cardiac arrest and cardiogenic shock impose extraordinary burdens on patients and caregivers, yet delivery of palliative care remains suboptimal with substantial gaps in evidence, practice, and access. Realizing palliative care's potential to enhance care will require rigorous research exploring effective and efficient delivery models, equitable access, standardized generalist palliative care education and training, and strategic specialist deployment to improve the lived experience of these patients and their caregivers.
